# Skin Grafting

**Published:** 1890-06-21

**Authors:** 


### THE PRACTITIONER'S MIRROR AND RETROSPECT.

### SURGERY.

### SKIN GRAFTING.

Some time ago ("Retrospect," February 15th) we noticed a case of transplantation of rabbit's conjunctiva to the human eye, reported by Dr. J. R. Wolfe, professor of ophthalmology at St. Mungo's College. Several other cases more or less successful, have occurred, so that the operation may now perhaps be regarded as an established one in ophthalmic surgery. Skin grafting is also now an established operation in general surgery, and we think it is not resorted to quite as frequently as it might be, although of course every ulceration is not suited to the treatment.

A case of extensive burn of the leg treated by grafting the skin of a dog has been recently reported by Dr. Alexander Miles, late resident surgeon at the Royal Infirmary, Edinburgh. The leg was scalded all round, from the middle of the patella to the ankle, the foot having been protected by the boot. A month afterwards it was found that the whole of the skin of the leg, from the level of the middle of the patella to the ankle, with the exception of a small island on the front of the tibia, had been completely destroyed. The greater part of the space was covered with granulations, but some few sloughs were not separated. These were got rid of by means of boracic lint poultices. As a sufficient quantity of human skin to cover the area could not be obtained, it was suggested that the skin of some animal should be used. A young greyhound puppy having been procured, and killed by chloroform, the anterior abdominal walls and flanks were shaven and the skin dissected off, leaving the subcutaneous fat. The boy's leg being well cleaned and all bleeding from granulations being arrested, the puppy's skin was applied in strips measuring six inches long and an inch broad, and pressed firmly into the ulcer in the long axis of the limb. The dressing consisted of small pieces of protective applied next the grafts, with the edges overlapping to facilitate removal, as well as escape of discharge. Outside, were placed a few layers of unprepared gauze moistened with weak boracic lotion, the whole being kept moist by gutta-percha tissue. The dressing was secured with cotton wool and a firm bandage. Three days after the operation it was found that one graft had sloughed, but the others were adherent. During the next fortnight healing went on rapidly. But a few small grafts of human skin taken from a boy were now used, and all did well. One or two spots remaining uncovered pieces of frog's skin were tried but failed. Seven months after the operation the leg was quite healed, and as useful as ever. There was no contraction, the colour of the skin is that of the normal surface, there is no evidence of development of hair, while sensation and temperature are the same as in the other leg. The author observes that there are three main factors upon which the success of such a case depends, viz., the general condition of the patient, the condition of the ulcer, and the means adopted to hasten the healing of the ulcer. In this case the patient was young, the ulcer a typical healthy granulating surface, and the covering adopted taken from a young animal. Dr. Miles regards this latter as a very important point. He remarks: "In the first few days of extra-uterine life, the creature grows very rapidly, and by grafting an area of young tissue with a potentially great developmental power, we quickly cover in the ulcerated area, and so prevent the contraction which invariably results after extensive burns, when these are allowed to heal without artificial aid."

Skin grafting was first practised by Reverdin, of Geneva, and introduced into this country by Pollock. Holmes says that a small piece of the surface, consisting of little more than the epithelial layer, should be planted not far from the edge of the sore, and that the growth of the graft seems to depend on the cells of the *rete malpighi*, and a combination of fine forceps and scissors has been devised for the purpose of taking up grafts. But in the case mentioned above, which resulted so successfully, all excepting subcutaneous fat was applied. Skin grafting has, perhaps, been practised more by American than by British surgeons. Dr. Lee, of Boston, has used lamb's skin extensively, and advises feeding the lambs for some time on milk before using the skin. Very successful cases have been reported of the treatment of ectropion and symblepharon by skin grafting. It would appear that the skin of almost any living thing will grow if properly applied to the human subject. Holmes speaks favourably of frog's skin, first recommended by Allen, of Durham. The application of epidermis scraped from the skin is not satisfactory. It should not be forgotten that a case of syphilitic infection by a graft taken from a person suffering from secondaries has been recorded. Essentials to success are fair general health of the patient and an unirritable sore. It is, therefore, evident that the process is not applicable to all ulcerations.

The process known as sponge grafting may also be noticed. This was first proposed by Professor Hamilton, of Aberdeen. The sponge is cut into thin pieces in a freezing microtome, and these are applied with moderate pressure on the granulating surface. When they appear to have become impregnated by the sprouting of granulations into the interstices of the sponge, fresh layers are applied till the cavity is filled up. The framework of the sponge appears to be organised in the same sense as carbolised ligatures are said to be organised, *i.e.*, its place is occupied ultimately by organised tissue. If too large pieces of sponge are used they retain discharge and retard healing. And if very small slices are used there is not sufficient substance to protect granulations, and really this is the only use of the sponge. Some years ago successful cases were reported from the Whitechapel Infirmary, but when tried at St. Thomas's Hospital it was not very successful.

